# Trends in Use and Comparison of Stereotactic Body Radiation Therapy, Brachytherapy, and Dose-Escalated External Beam Radiation Therapy for the Management of Localized, Intermediate-Risk Prostate Cancer

**DOI:** 10.1001/jamanetworkopen.2020.17144

**Published:** 2020-09-24

**Authors:** Kevin A. Nguyen, Alan Lee, Sagar A. Patel, Arun Chakravorty, James B. Yu, Amar U. Kishan, Albert J. Chang

**Affiliations:** 1 Department of Radiation Oncology, David Geffen School of Medicine, University of California, Los Angeles; 2 Department of Radiation Oncology, Emory University, Atlanta, Georgia; 3 Deparment of Radiation Oncology, Yale School of Medicine, New Haven, Connecticut

## Abstract

This cohort study examines trends in the use of brachytherapy, external beam radiotherapy, and stereotactic body radiation therapy for disease management in patients with localized prostate cancer and compares outcomes between these therapies.

## Introduction

Whereas brachytherapy (BT) and dose-escalated external beam radiotherapy (DE-EBRT) have longstanding use in the treatment of patients with localized prostate cancer (CaP), stereotactic body radiation therapy (SBRT) is an emerging option owing to cost effectiveness, patient convenience, and noninferior tumor control and acute toxic effects.^1,2^ The increase in radiation options and paucity of comparative evidence present challenges in guiding patient-centered care.^3^ Using the National Cancer Database, we compared use and outcomes between SBRT, BT, and EBRT for the treatment of patients with intermediate risk CaP.

## Methods

### Patient Population

For this cohort study, patients were identified in the National Cancer Database who had National Comprehensive Cancer Network intermediate risk CaP (Gleason score of 6-7, clinical stage T1-T2, and prostate-specific antigen <20 ng/mL [to convert to micrograms per liter, multiply by 1.0]) diagnosed between January 1, 2004, and December 31, 2014. For EBRT, only ≥75 Gy or ≥42 fractions of treatment were included. Stereotactic body radiation therapy was defined as 5 fractions of ≥7 Gy per fraction. The University of California, Los Angeles granted institutional review board exemption for the use of a deidentified national database. This study followed the Strengthening the Reporting of Observational Studies in Epidemiology (STROBE) reporting guideline.

Data were analyzed from February 1 to March 1, 2020. Cox proportional hazards were calculated to assess factors independently associated with overall survival (OS). To account for potential confounders, propensity score matching was performed in a 1:1 manner, and the distribution of propensity scores in matched cohorts were then verified (eFigure in the Supplement). Two-sided *P* values were calculated using the log-rank test, and statistical significance was considered if *P* ≤ .05. Statistical analysis was performed using JMP, version 11.2.1 (SAS Institute Inc) and R, version 4.0.2 (R Project for Statistical Computing) (MatchIt package, version 3.3.0).

## Results

Overall, 30 766 men (median age at diagnosis, 69 years [interquartile range, 63-74 years]) were eligible for analysis: 24 953 (81.1%) had favorable intermediate risk, and 5813 (18.9%) had unfavorable intermediate risk. A total of 12 864 patients (41.8%) received BT, 17 247 (56.1%) received DE-EBRT, and 655 (2.1%) received SBRT (Table). From 2004 to 2014, SBRT use (0.03% to 10.6%) and DE-EBRT use (48.3% to 62.0%) steadily increased, with a corresponding decline in BT use (48.3% to 27.4%) (Figure).

**Table.** Comparison of Clinicopathologic Characteristics by Treatment Status

Variable	No. (%)			*P* value
	BT	DE-EBRT	SBRT	
No. of patients	12 864 (41.8)	17 247 (56.1)	655 (2.1)	
Facility type				
Academic	4392 (34.1)	7875 (45.7)	433 (66.1)	<.001
Community	8472 (65.9)	9372 (54.3)	222 (33.9)	
Age of onset, median (IQR), y	68 (62-73)	70 (64-75)	69 (64-74)	<.001
Distance to treatment center, median (IQR), miles	11.6 (5.1-28.2)	8 (3.7-18.7)	11.6 (5.5-29.3)	<.001
Insurance status				
Medicaid	186 (1.5)	413 (2.4)	13 (2.0)	<.001
Medicare	7180 (55.8)	10 657 (61.8)	433 (66.1)	
Other government	205 (1.6)	439 (2.6)	<10^a^	
Private insurance	5034 (39.1)	5198 (30.1)	181 (1.1)	
Uninsured	89 (0.7)	240 (1.4)	<10^a^	
Unknown status	170 (1.3)	300 (1.7)	18 (2.8)	
Median income, quartiles, US$				
<38 000	2113 (16.4)	2924 (17.0)	73 (11.2)	<.001
38 000-47 999	3131 (24.3)	3742 (21.7)	88 (13.4)	
48 000-62 000	3322 (25.8)	4613 (26.8)	132 (20.2)	
>63 000	4119 (32.0)	5757 (33.4)	353 (53.9)	
Unknown	179 (1.4)	211 (1.2)	<10^a^	
Charlson/Deyo score				
0	11 159 (86.8)	15 334 (88.9)	546 (83.4)	<.001
1	1470 (11.4)	1585 (9.2)	97 (14.8)	
2	235 (1.8)	328 (1.9)	12 (1.8)	
Race/ethnicity				
Black	1601 (12.5)	2681 (15.5)	89 (13.6)	<.001
Asian	254 (2.0)	356 (2.1)	<10^a^	
White	10 751 (83.6)	13 872 (80.4)	550 (84.0)	
Other/unknown^b^	258 (2.0)	338 (2.0)	<10^a^	
NCCN risk classification				
Favorable intermediate	11 032 (85.8)	13 377 (77.6)	544 (83.1)	<.001
Unfavorable intermediate	1832 (14.2)	3870 (22.4)	111 (17.0)	
Brachytherapy dosing				
HDR	2901 (22.6)	NA	NA	NA
LDR	5670 (44.1)	NA	NA	
NOS	4293 (33.4)	NA	NA	

Abbreviations: BT, brachytherapy; DE-EBRT, dose-escalated external beam radiotherapy; HDR, high dose rate; IQR, interquartile range; LDR, low dose rate; NA, not applicable; NCCN, National Comprehensive Cancer Network; NOS, not otherwise specified; SBRT, stereotactic body radiation therapy.

^a^ To prevent patient identifiability, the National Cancer Database does not allow reporting of any cell sizes of fewer than 10 patients.

^b^ Includes Pacific Islander, Southeast Asian, unknown, or not otherwise specified.

**Figure.**
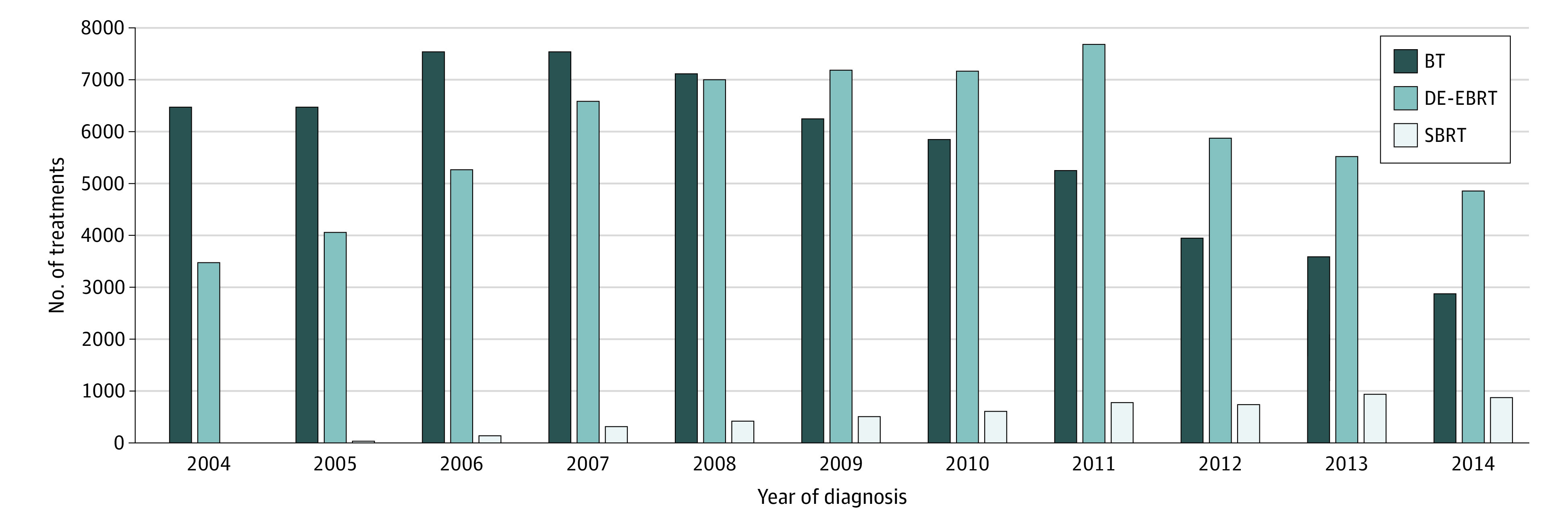
Use of Treatments by Year of Diagnosis From 2004 to 2014

The median follow-up was 6.7 years (range, 0-11.9 years). In the favorable intermediate risk cohort, there was no significant OS difference in pairwise comparisons of BT vs SBRT (HR, 0.804; 95% CI, 0.593-1.09; *P* = .16; 10-year OS, 67.02% vs 64.2%) or SBRT vs DE-EBRT (HR, 1.096; 95% CI, 0.810-1.48; *P* = .55; 10-year OS, 64.2% vs 70.9%). Men receiving BT had a small but statistically significant improvement in OS compared with those receiving DE-EBRT (HR, 0.881; 95% CI, 0.829-0.938; *P* < .001; 10-year OS, 69.8% vs 66.1%).

In the unfavorable intermediate risk cohort, there were no OS differences in pairwise comparisons between BT and SBRT (HR, 0.749; 95% CI, 0.419-1.34; *P* = .33; 10-year OS, 64.9% vs 63.2%) and between SBRT and DE-EBRT (HR, 1.36; 95% CI, 0.746-2.69; *P* = .32; 10-year OS, 63.2% vs 66.6%). Men receiving BT demonstrated a small but statistically significant improvement in OS compared with those receiving DE-EBRT (HR, 0.818; 95% CI, 0.716-0.936; *P* < .001; 10-year OS, 61.2% vs 58.7%).

## Discussion

This cohort study is a preliminary evaluation comparing outcomes of patients with intermediate-risk CaP, with results suggesting no difference in long-term survival between patients treated with SBRT, EBRT, or BT. These findings corroborate the results of the Hypofractionated Radiotherapy of Intermediate Risk Localised Prostate Cancer (HYPO-RT-PC) study demonstrating noninferior 5-year failure-free survival between ultrahypofractionated and conventionally fractionated therapy.^1^ Although BT has long been shown to be both clinically effective and cost-effective in the management of localized CaP, many studies, including the present study, show a decline in use over the last decade.^4^ As radiation modalities trend toward hypofractionation with major considerations toward cost-effective treatment, our preliminary evaluation suggests that SBRT and BT remain appropriate management strategies in delivering value-based care.

This study has some limitations. The retrospective nature involving a national database includes potential selection bias and unbalanced variables between treatment groups that were not accounted for despite propensity score matching. Furthermore, parameters such as toxicity, biochemical recurrence, distant recurrence, and cancer-specific survival could not be assessed in the National Cancer Database.

## Notes

Supplement. **eFigure.** Comparison of Distribution of Propensity Scores and Standardized Mean Differences Before and After Matching
