# Supplementary material for: Trends in Use and Comparison of Stereotactic Body Radiation Therapy, Brachytherapy, and Dose-Escalated External Beam Radiation Therapy for the Management of Localized, Intermediate-Risk Prostate Cancer
Source: JAMA Netw Open. 2020 Sep 24;3(9):e2017144. doi: 10.1001/jamanetworkopen.2020.17144 (PMC7516602; doi:10.1001/jamanetworkopen.2020.17144)
Supplement: Supplement. — eFigure. Comparison of Distribution of Propensity Scores and Standardized Mean Differences Before and After Matching [file jamanetwopen-e2017144-s001.pdf]

Supplementary Online Content

Nguyen KA, Lee A, Patel SA, et al. Trends in use and comparison of stereotactic body radiation therapy, brachytherapy, and dose-escalated external beam radiation therapy for the management of localized, intermediate-risk prostate cancer. *JAMA Netw Open*. 2020;3(9):e2017144. doi:10.1001/jamanetworkopen.2020.17144

eFigure. Comparison of Distribution of Propensity Scores and Standardized Mean Differences Before and After Matching

This supplementary material has been provided by the authors to give readers additional information about their work.

© 2020 Nguyen KA et al. *JAMA Network Open.*

eFigure: Comparison of Distribution of Propensity Scores and Standardized Mean Differences Before and After Matching: NCCN favorable intermediate risk for **a):** BT vs. SBRT, **b):** DE-EBRT vs. SBRT, **c):** DE-EBRT vs. BT; NCCN unfavorable intermediate risk for **d):** BT vs. SBRT, **e):** DE-EBRT vs. SBRT, **f):** DE-EBRT vs. BT

A

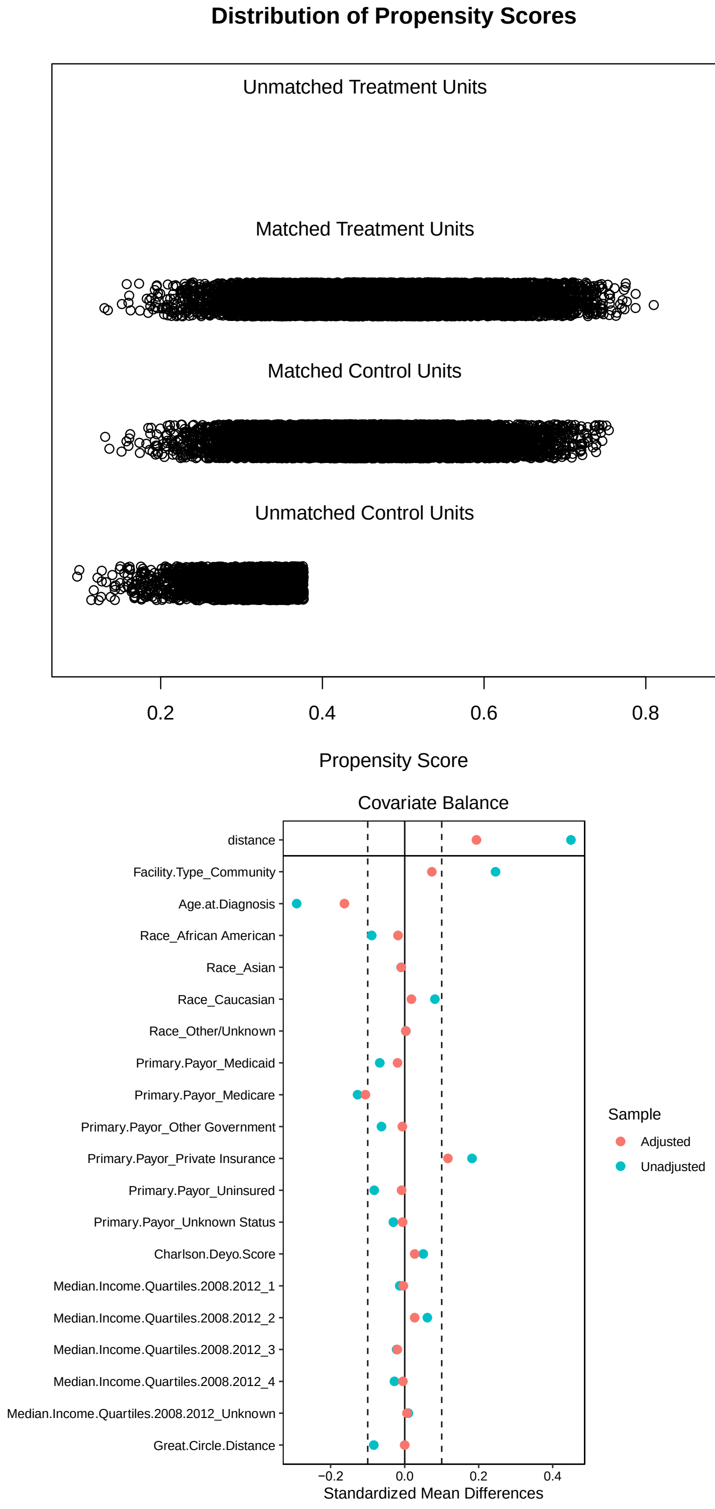

© 2020 Nguyen KA et al. *JAMA Network Open.*

B

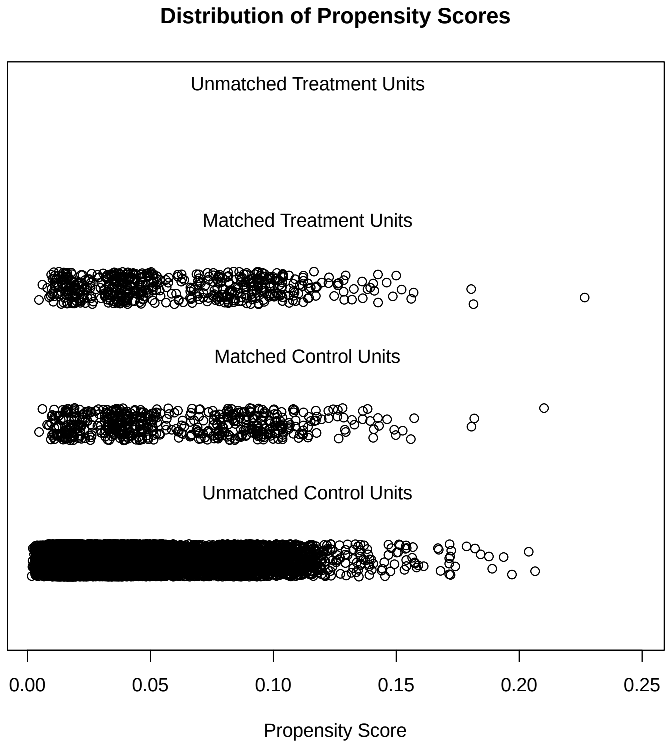

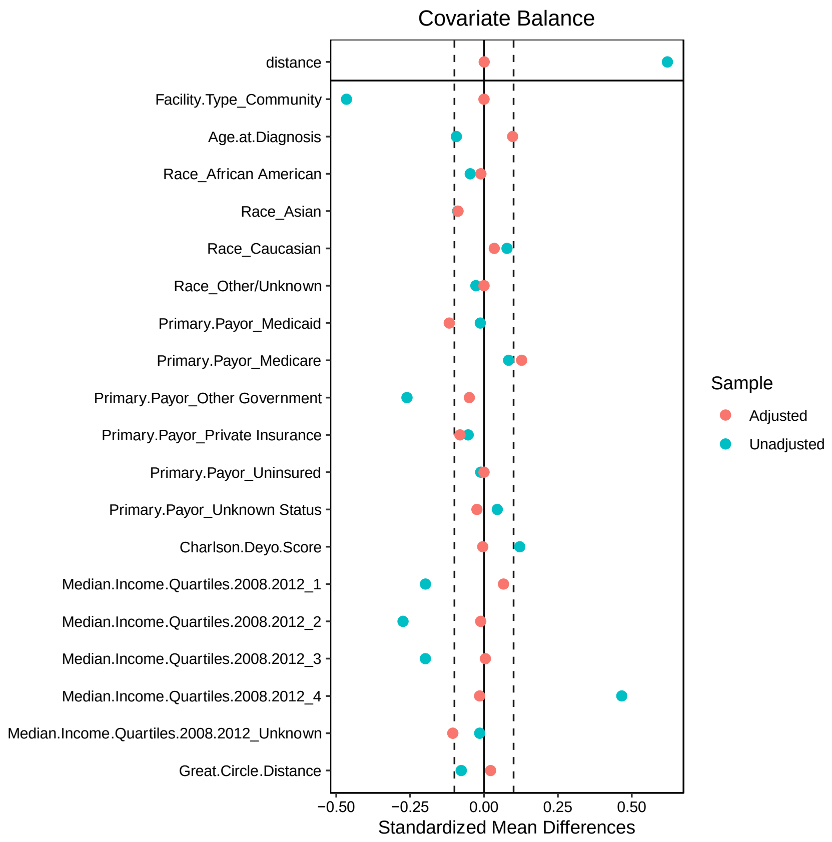

© 2020 Nguyen KA et al. *JAMA Network Open.*

C

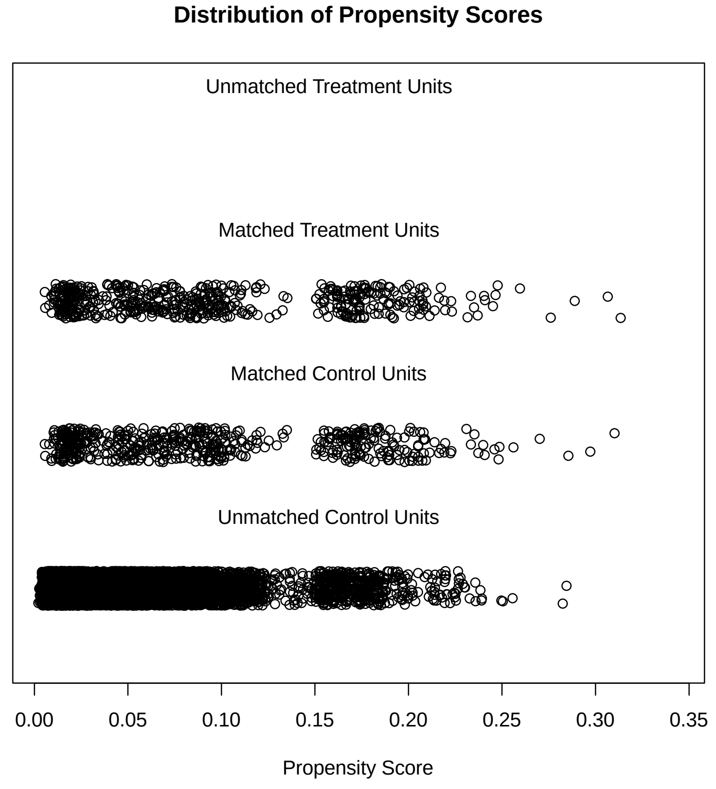

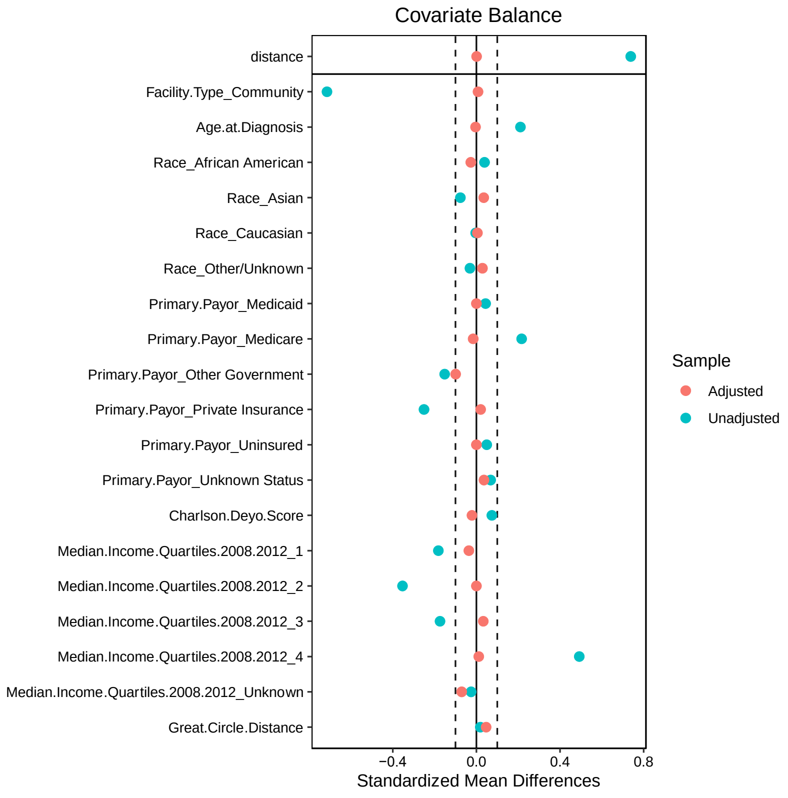

© 2020 Nguyen KA et al. *JAMA Network Open.*

D

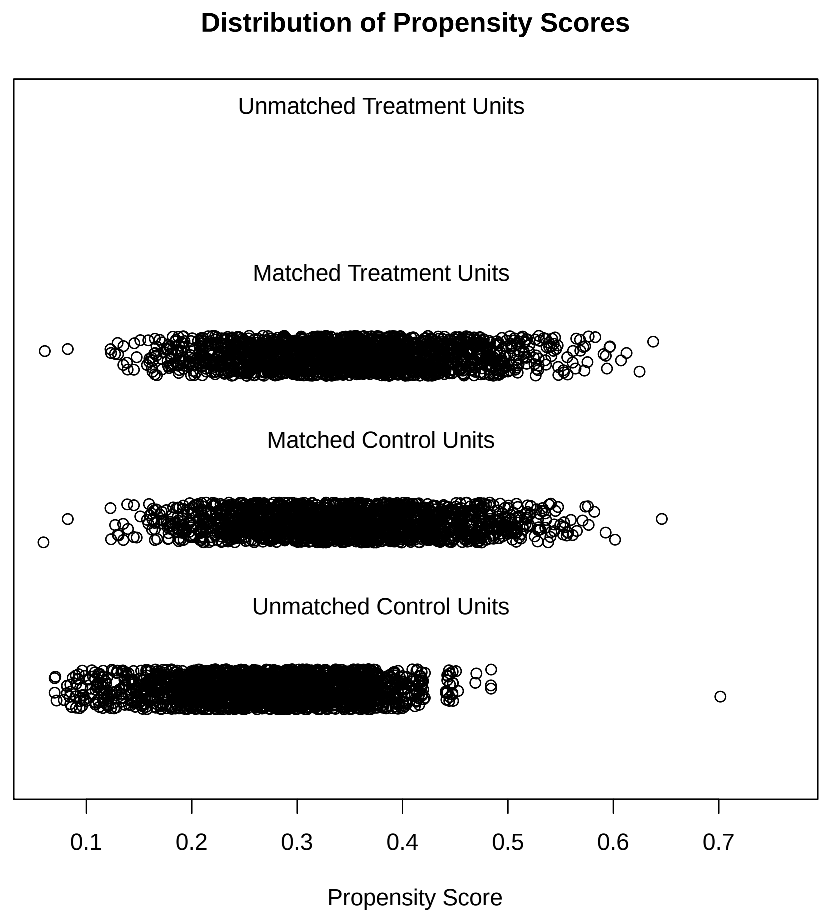

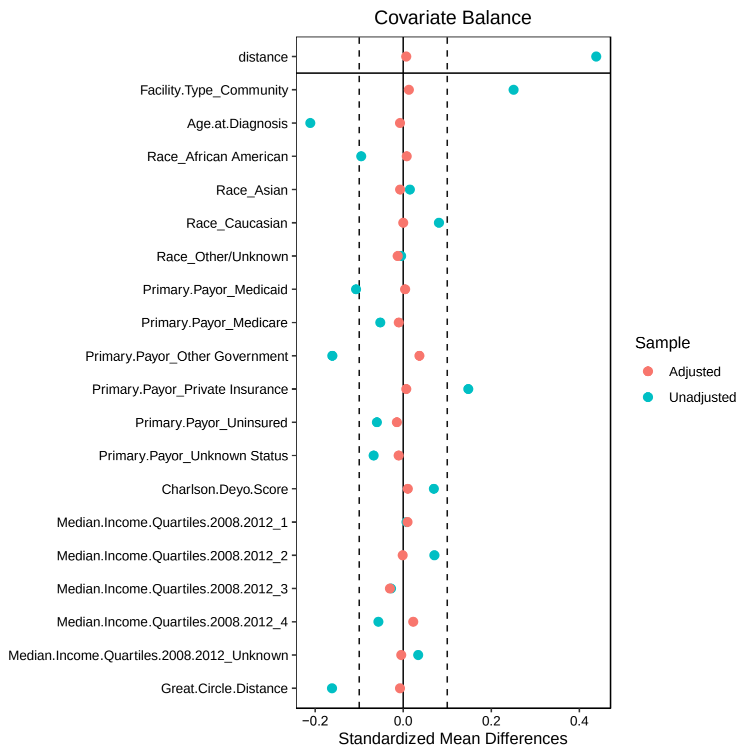

© 2020 Nguyen KA et al. *JAMA Network Open.*

E

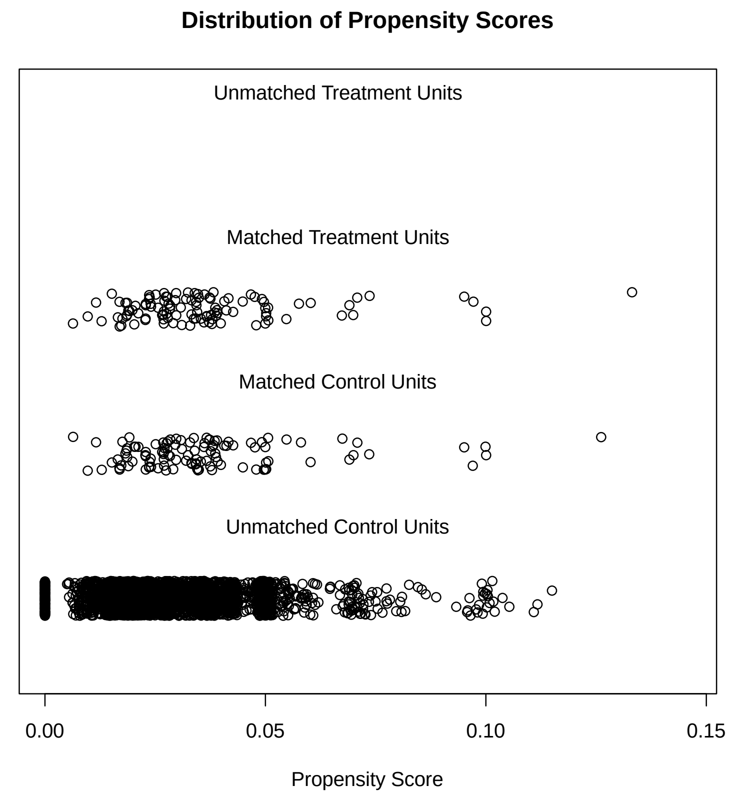

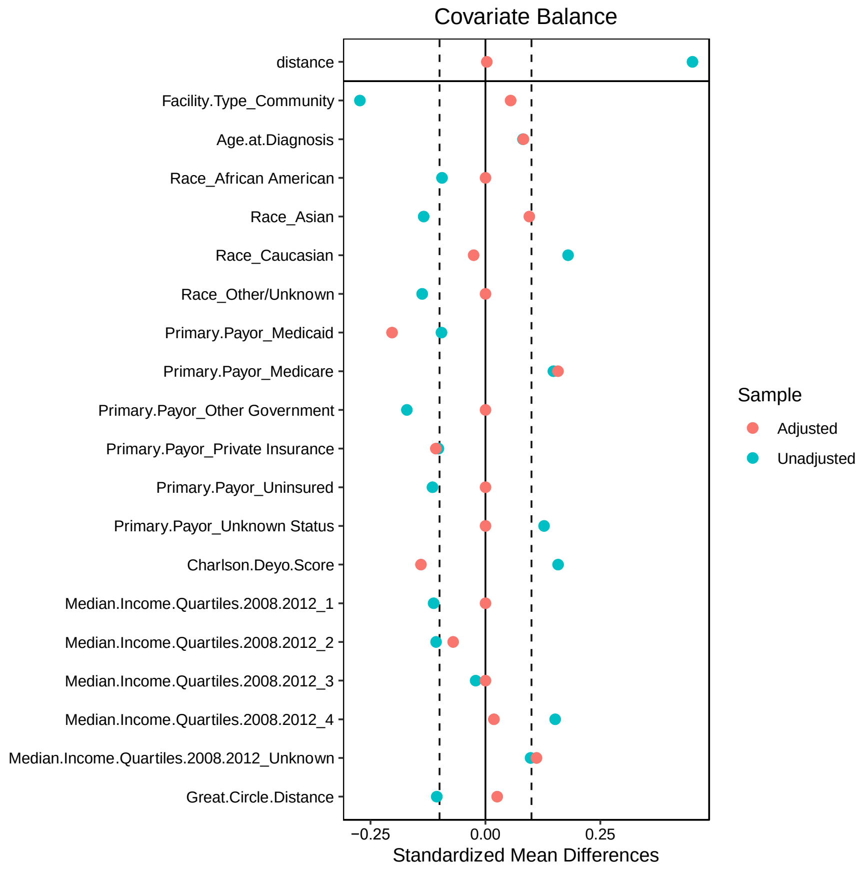

© 2020 Nguyen KA et al. *JAMA Network Open.*

F

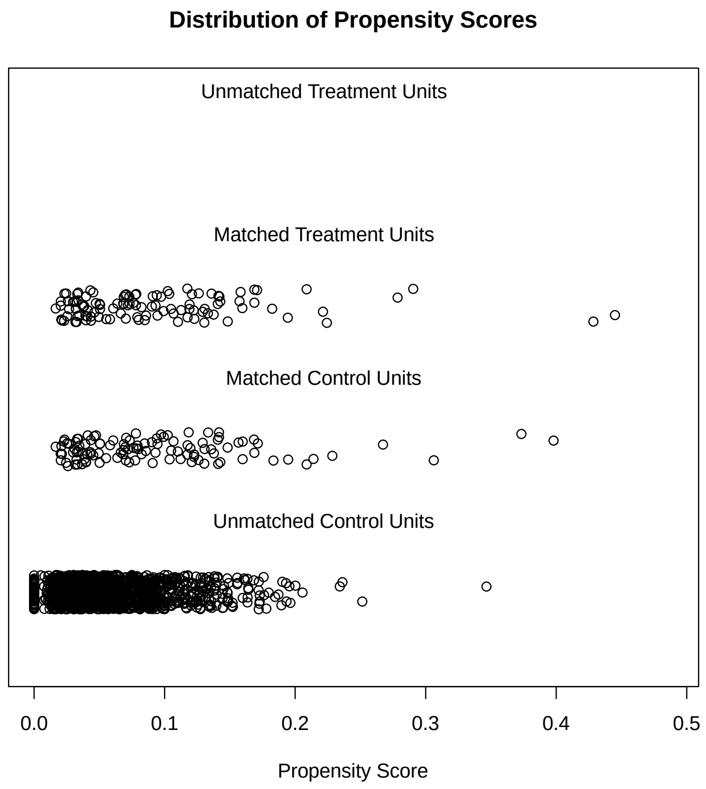

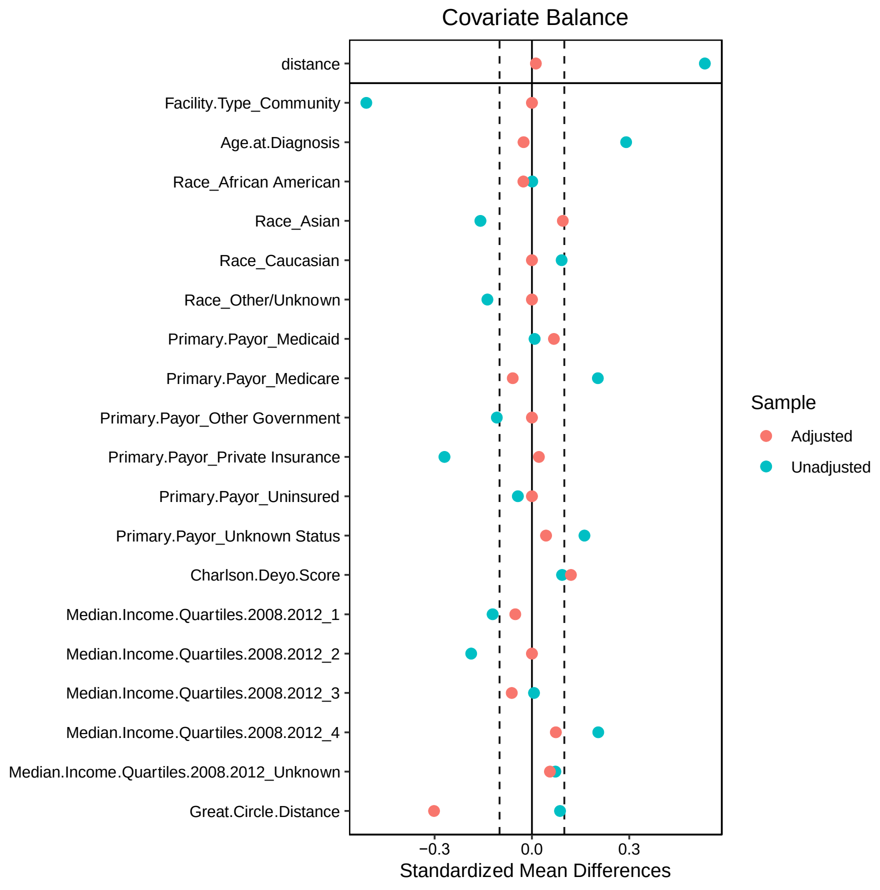

© 2020 Nguyen KA et al. *JAMA Network Open.*
